# Methodological quality of Campbell Systematic Reviews has improved over the past decade

**DOI:** 10.1002/cl2.1358

**Published:** 2023-09-26

**Authors:** Yanfei Li, Omar Dewidar, Xiaoqin Wang, Elizabeth Ghogomu, Arpana Wadhwani, Ke Guo, Mina Ma, Victoria Barbeau, Bei Pan, Leenah Abdelrazeq, Zijun Li, Amjad Alghamyan, Liping Guo, Fatima Jahel, Junjie Ren, Mohamad Tarek Madani, Sarah Allam, Tarannum Hussain, Minyan Yang, Waleed Kojan, Xiuxia Li, Kehu Yang, Vivian Welch

**Affiliations:** ^1^ School of Basic Medical Sciences, Evidence Based Medicine Center Lanzhou University Lanzhou China; ^2^ Bruyère Research Institute University of Ottawa Ottawa Canada; ^3^ University of Ottawa Heart Institute University of Ottawa Ottawa Canada; ^4^ Evidence Based Social Science Research Center/Health Technology Assessment Center, School of Public Health Lanzhou University Lanzhou China; ^5^ School of Health Sciences Carleton University Ottawa Canada; ^6^ University of Toledo College of Medicine and Life Sciences Toledo Ohio USA

The Campbell Collaboration was established in 2001 to promote positive social and economic change through supporting the conduct of high‐quality systematic reviews and promoting their use in decision making (Welch, 2018). Wang et al. (2021) found that the methodological quality of Campbell reviews of intervention effectiveness published between 2011 and 2018 improved over time, and particularly after the introduction of the 2014 Methodological Expectations for Conducting Campbell Intervention Reviews (MECCIR) (Wang et al., 2021). For the 96 systematic reviews published between 2011 and 2018, the methodologic quality as assessed by the AMSTAR tool was 16 (17%) reviews rated as high quality, 40 (42%) as moderate, 24 (25%) as low, and 16 (17%) as critically low (Wang et al., 2021).

Based on this assessment, Campbell provided feedback to all editorial teams on the quality of reviews and areas for improvement. We decided to conduct a follow‐up analysis to evaluate the quality of Campbell reviews published since 2018 and compare the findings with the baseline assessment to identify areas where improvements are still needed.

We conducted the quality assessment of Campbell systematic reviews of intervention effectiveness published in the past 5 years (February 2018 to November 2022) using the AMSTAR 2.0 tool (Shea et al., 2017). A total of 77 intervention reviews were included. All analyses were conducted using R software.

Regarding the overall methodological quality of the Campbell reviews from 2018 to 2022, 39% were high, 16% moderate, 27% low and 18% critically low quality (Figure 1). Twelve of the 16 AMSTAR 2.0 items were completely or partially addressed in more than 80% of the reviews. The following three items were addressed in less than 70% of the reviews (Supporting Information: Figure 1):

- Sources of funding for the included studies (34%, 26) (AMSTAR item 10).
- Assessed potential impact of risk of bias in individual studies on the results of the meta‐analysis or other evidence synthesis (52%, 40) (AMSTAR item 12).
- List of excluded studies with justifications (60%, 46) (AMSTAR item 7).

**Figure 1:**
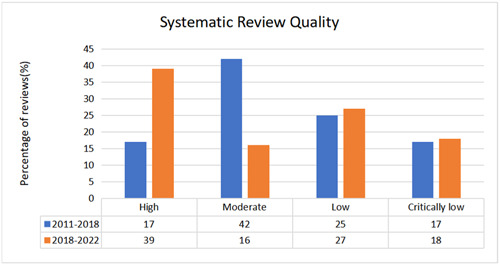
Overall methodological quality of Campbell systematic reviews.

Compared with the reviews published before 2018, the overall methodological quality of the recent reviews has generally improved (Figure 1). The proportion of high‐quality reviews has doubled (17% to 39%), while the proportion of moderate quality reviews has been reduced by more than half (42% to 16%). However, there was little difference in the percentage of reviews rated as low (25% vs. 27%) and critically low (17% vs. 18%).

Since the baseline assessment of Campbell reviews published between 2011 and 2018, some reporting deficiencies have improved and are now reported in over 70% of the reviews. The factors that improved were justifying the choice of eligible study designs, explaining heterogeneity in results, and discussing the impact of publication bias (Figure 2). However, reporting the source of funding and the impact of risk of bias in individual studies on the results of the meta‐analysis were persistently inadequately considered but more frequently observed in the last 5 years (15% to 34%, and 33% to 52%, respectively). Of note, fewer reviews in the last 5 years reported the list of excluded studies with justifications than did the sample of 2011–2018 (92% to 60%). This is a critical flaw in the AMSTAR scale that leads to lower quality ratings.

**Figure 2:**
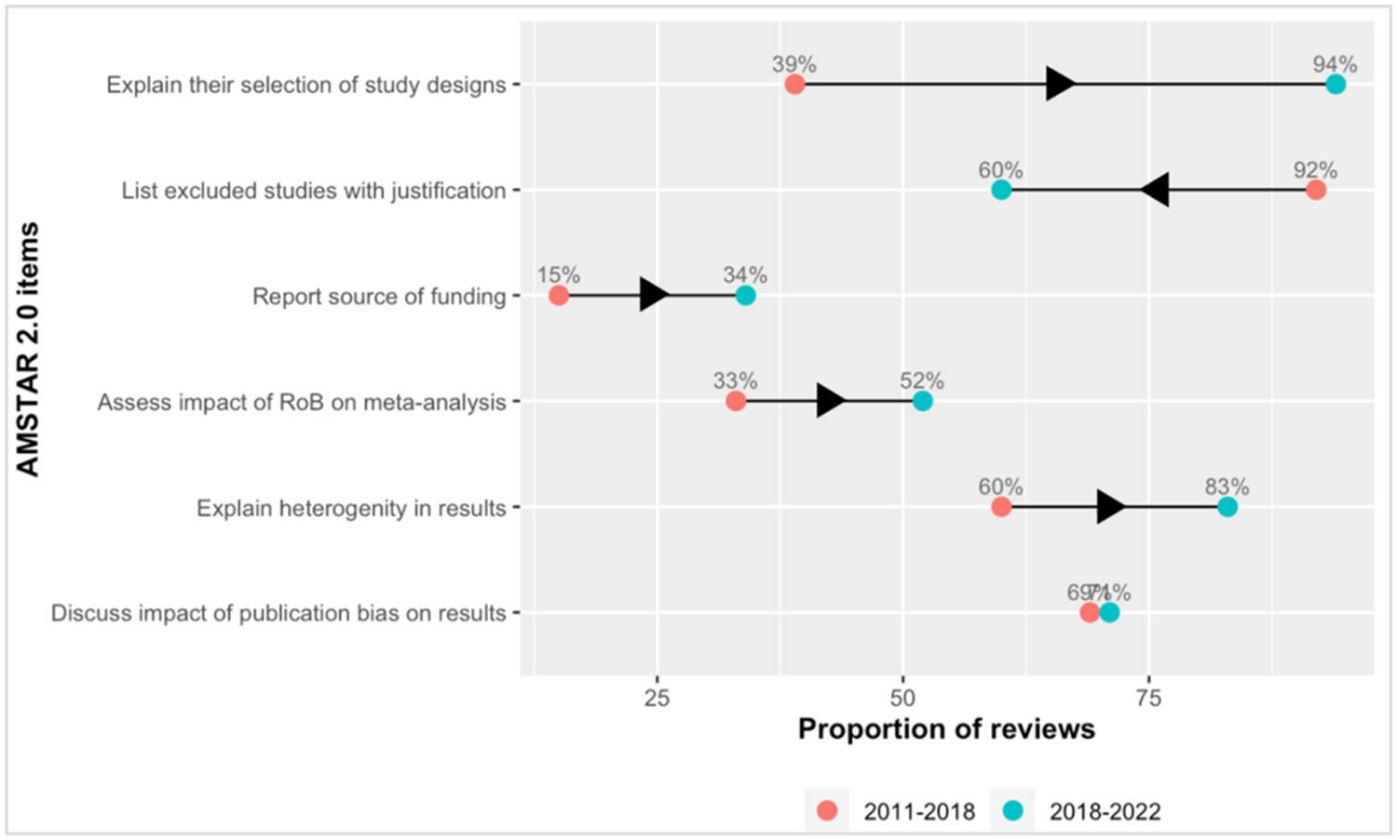
Changes in reporting of AMSTAR 2.0 items reported in less than 70% of Campbell reviews in 2011–2018.

Although there has been continuous improvement in the quality of Campbell reviews, there is a need to improve reporting of excluded studies, sources of funding for studies, impact of risk of bias on the meta‐analysis, and assessing impact of publication bias.

To address these shortcomings, the Campbell editorial board has implemented three strategies going forward and will monitor the quality of reviews annually.

First, all Campbell authors have access to RevmanWeb for authoring their Campbell reviews and evidence and gap maps. Campbell's template for reviews of intervention effectiveness have been modified to mention each of the 16 AMSTAR items in the guidance for authors as they write their reviews. This aims to raise awareness of items that influence methodological quality during the conduct of the review.

Second, an internal Campbell editor assesses each Campbell review before sending for external review. Campbell has included the AMSTAR items in the internal editorial checklists and feedback forms. This will help editors to assess if all AMSTAR items are reported and provide feedback to authors during the editorial process.

Third, although implementation of the MECCIR expectations led to improved quality from 2014 to 2018, the checklists are burdensome for both authors and editors (with 79 items in the MECCIR for conduct and 102 items in the MECCIR for reporting). Campbell is currently updating MECCIR to create a unified checklist with the goal of making it easier for authors and editors to ensure that methodologic standards are met. Furthermore, this updated guidance aims to include all relevant items of AMSTAR and PRISMA 2020 (Page et al., 2021) in this unifies checklist. This updated guidance will be available by Fall 2023.

Systematic reviews have a special importance for decision making. They aim to summarize the best available evidence on a specific research question to inform practice guidelines and reveal knowledge gaps to guide future research initiatives in a wide range of sectors (Collaboration, 2018; Li et al., 2021; Yang, Li, & Bai, 2018). The trustworthiness of a systematic review depends on its methodological rigor and reporting quality (Pussegoda et al., 2017).

We welcome feedback on these measures to continuously improve the quality of Campbell systematic reviews.

## Supporting information

Supporting information.
