# CORRIGENDUM

**DOI:** 10.1002/cl2.1384

**Published:** 2024-03-05

We are writing to correct our editorial on AMSTAR 2.0 quality of Campbell systematic reviews. Upon receiving feedback from Campbell authors on the rating of their reviews, we found that our operationalization of the AMSTAR 2.0 tool was overly strict for two items: (1) listing the excluded studies and providing justification for their exclusion (item 7), and (2) investigation of publication bias and discussing its likely impact on the review findings (item 15). Both of those items are critical items which significantly affects the overall quality rating. We consulted with the lead author of AMSTAR2 (Bev Shea) and the detailed AMSTAR2 guidance and we reevaluated all the reviews which were rated as not meeting these two criteria.

For item 7: listing the excluded studies and providing justification for their exclusion, we initially required a table with all of the excluded studies and reasons for exclusion to rate this as “yes.” On re‐evaluation, we identified reviews which reported a list of “near misses” (Higgins et al., 2023; The Methods Group of the Campbell Collaboration, 2017) or examples of excluded studies with rationales for exclusion in the text. We adjusted those reviews to “yes” if all studies were listed and “partial yes” if excluded studies were partially listed.

For item 15: investigation of publication bias and discussing its likely impact on the review findings, we required that reviews described how they assessed publication bias **and** described the implications on review findings in the discussion to rate this as “yes”. Reviews where the method was described, but its implications on the findings were not discussed explicitly, were rated as “no.” This criteria does not have a “partial yes” option. On discussion with Bev Shea, we decided that if a method for assessing publication bias is described and the discussion comments on issues related to publication bias such as comprehensiveness of searches, this justifies a “yes.” We adjusted the rating of those reviews which meet these criteria to “yes,” even if there was no explicit mention of publication bias in the discussion. For reviews with no quantitative meta‐analysis, this item was rated “not applicable” as per AMSTAR2 guidance.

**The corrected sections are shown below:**

1. The following updates were made in page one (paragraph 4): Regarding the overall methodological quality of the Campbell reviews from 2018 to 2022, **44%** were high, **17%** moderate, **29%** low and **10%** critically low quality (Figure 1). Twelve of the 16 AMSTAR 2.0 items were completely or partially addressed in more than 80% of the reviews. The following **two** items were addressed in less than 70% of the reviews (Supplementary Figure 1):
  - Sources of funding for the included studies (**35%, 27**) (AMSTAR item 10).
  - Assessed potential impact of risk of bias in individual studies on the results of the meta‐analysis or other evidence synthesis (**49%, 38**) (AMSTAR item 12).
2. The following updates were made in page one (paragraph 5): Compared to the reviews published before 2018, the overall methodological quality of the recent reviews has generally improved (Figure 1). The proportion of high‐quality reviews has doubled (17% to **44%**), while the proportion of moderate quality reviews has been reduced by more than half (42% to **17%**). However, there was little difference in the percentage of reviews rated as low (25% vs. **29%**) and critically low (17% vs. **10%**).
3. In the page one (paragraph 6), we updated the sentence “However, reporting the source of funding and the impact of risk of bias in individual studies on the results of the meta‐analysis were persistently inadequately considered but more frequently observed in the last 5 years (15% to **35%**, and 33% to **49%**, respectively).” and **deleted** the sentence “Of note, fewer reviews in the last 5 years reported the list of excluded studies with justifications than did the sample of 2011‐2018 (92% to 60%). This is a critical flaw in the AMSTAR scale that leads to lower quality ratings.”
4. In page one, paragraph 7, we deleted the “**excluded studies**.”
5. We have updated Figure 1, Figure 2, and **Supplementary Figure** 1.

**Figure 1:**
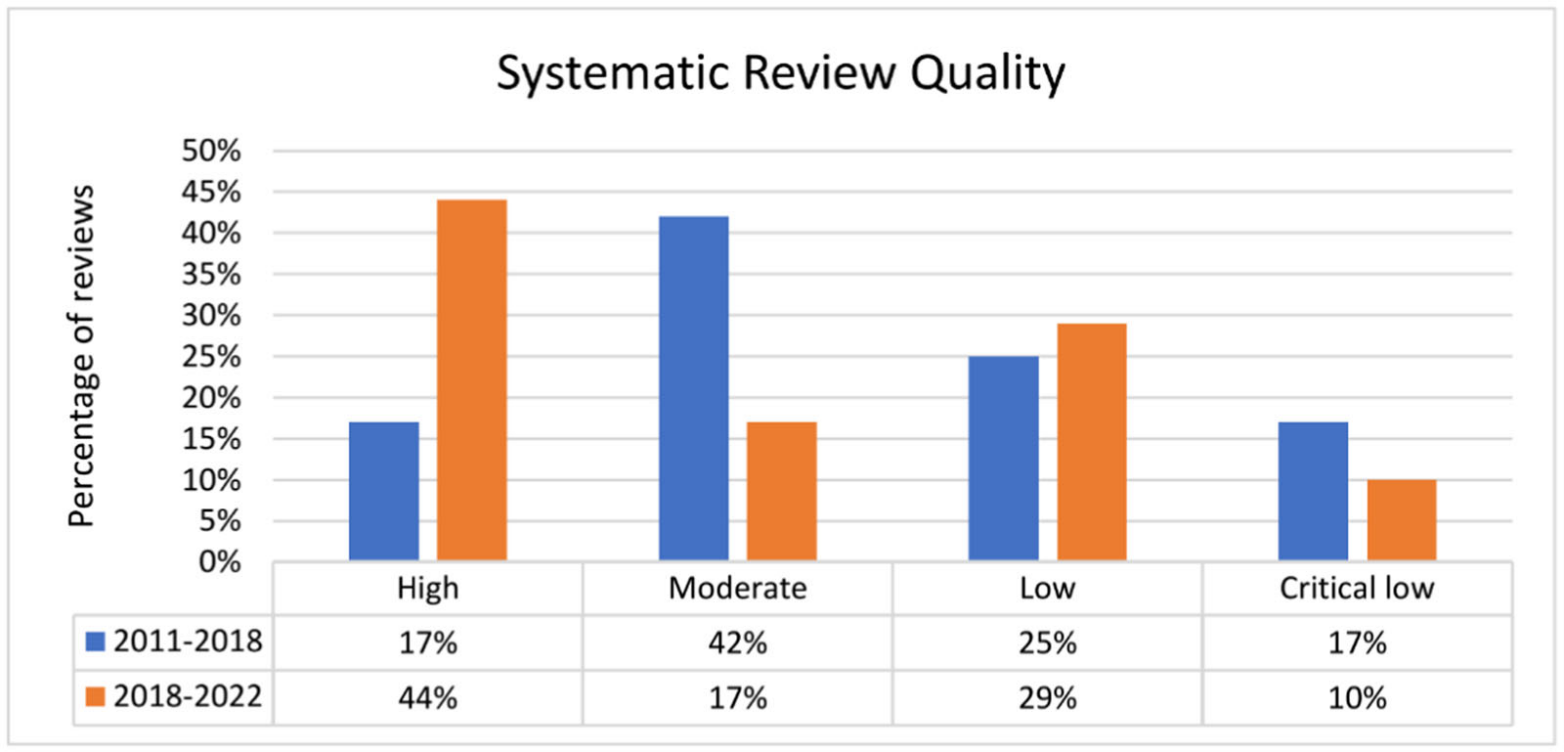
Overall methodological quality of Campbell systematic reviews.

**Figure 2:**
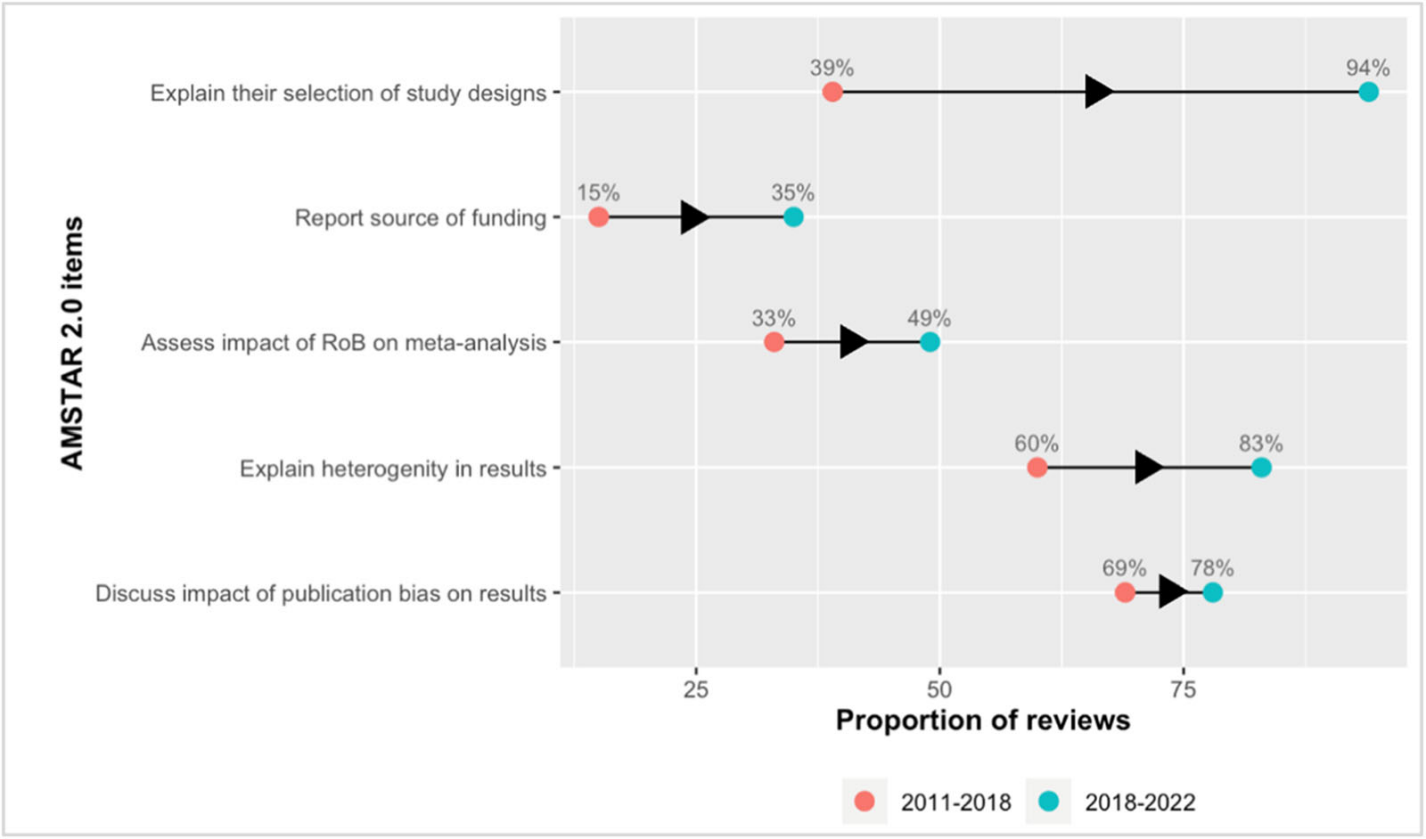
Changes in reporting of AMSTAR 2.0 items reported in less than 70% of Campbell reviews in 2011–2018.

## Supporting information

Supporting information.
