# Supplementary material for: CORRIGENDUM
Source: Campbell Syst Rev. 2024 Mar 5;20(2):e1384. doi: 10.1002/cl2.1384 (PMC10912977; doi:10.1002/cl2.1384)
Supplement: Supplementary file 1 — Supporting information. [file CL2-20-e1384-s001.docx]

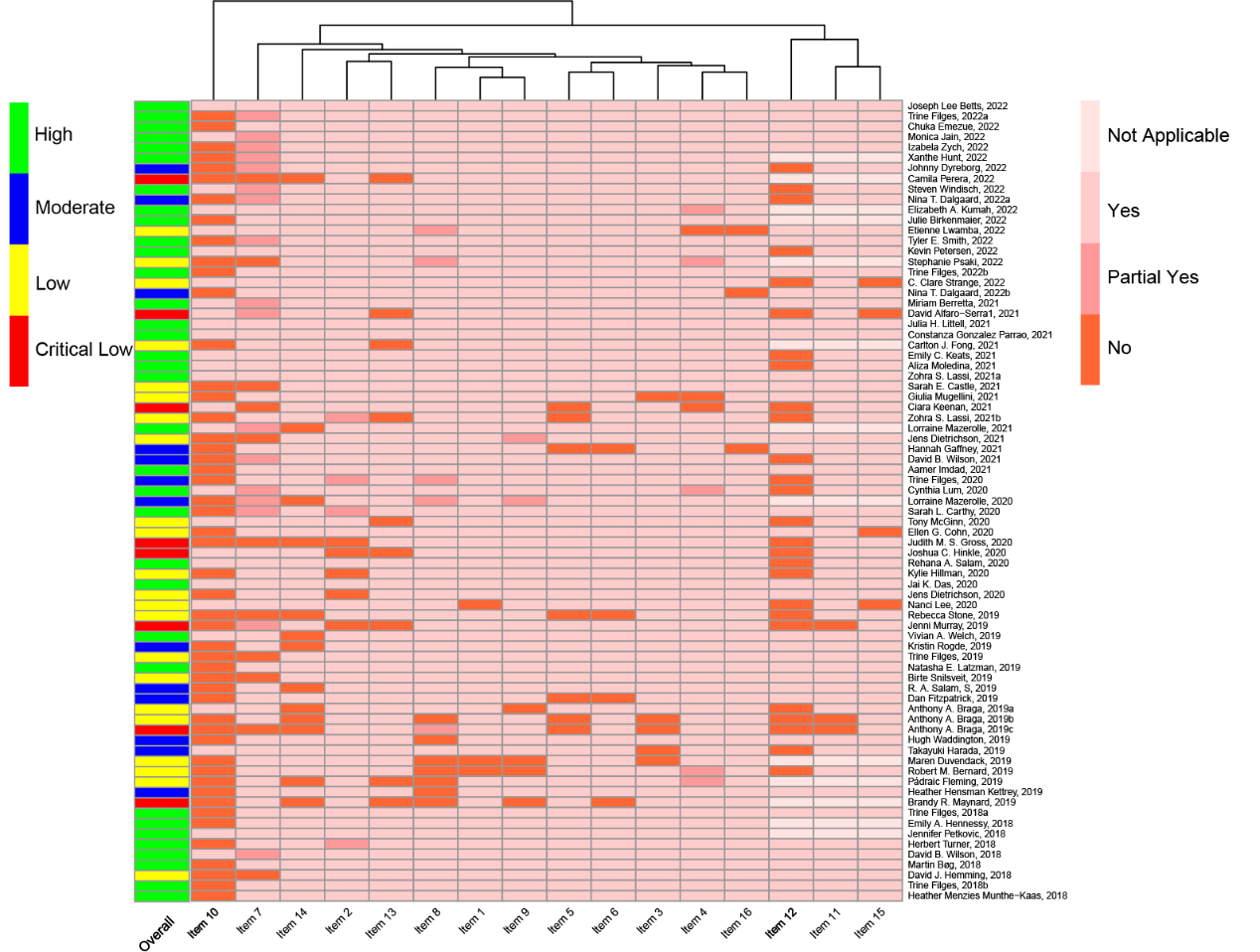


**Supplementary Figure 1.** Methodological quality assessment of 77 Campbell systematic reviews, published between February 2018 to Nov 2022. The items are ordered in terms of frequency of reporting of each AMSTAR item, with the least reported item on the left.
